# Body mass index modulates the relationship of sugar-sweetened beverage intake with serum urate concentrations and gout

**DOI:** 10.1186/s13075-015-0781-4

**Published:** 2015-09-22

**Authors:** Nicola Dalbeth, Amanda Phipps-Green, Meaghan E. House, Gregory D. Gamble, Anne Horne, Lisa K. Stamp, Tony R. Merriman

**Affiliations:** Department of Medicine, Faculty of Medical and Health Sciences, University of Auckland, 85 Park Road, Grafton, Auckland 1023 New Zealand; Department of Biochemistry, Division of Health Sciences, University of Otago, 710 Cumberland Street, Dunedin, 9016 New Zealand; Department of Medicine, Division of Health Sciences, University of Otago, 2 Riccarton Avenue, Christchurch, 8011 New Zealand

## Abstract

**Introduction:**

Both sugar-sweetened beverage (SSB) intake and body mass index (BMI) are associated with elevated serum urate concentrations and gout risk. The aim of this study was to determine whether the associations of SSB intake with serum urate and gout are moderated by BMI.

**Method:**

The effects of chronic SSB intake on serum urate and gout status were analysed in a large cross-sectional population study. The effects of an acute fructose load on serum urate and fractional excretion of uric acid (FEUA) were examined over 180 minutes in a short-term intervention study. In all analyses, the responses were compared in those with BMI <25 mg/kg^2^ (low BMI) and ≥25 mg/kg^2^ (high BMI).

**Results:**

In the serum urate analysis (n = 12,870), chronic SSB intake was associated with increased serum urate in the high BMI group, but not in the low BMI group (*P*_difference_ = 3.6 × 10^−3^). In the gout analysis (n = 2578), chronic high SSB intake was associated with gout in the high BMI group, but not in the low BMI group (*P*_difference_ = 0.012). In the acute fructose loading study (n = 76), serum urate was increased in the high BMI group at baseline and throughout the observation period (P_BMI group_ <0.0001), but there were similar acute serum urate increases in both BMI groups in response to the fructose load (*P*_interaction_ = 0.99). The baseline FEUA was similar between the two BMI groups. However, following the fructose load, FEUA responses in the BMI groups differed (*P*_interaction_ <0.0001), with increased FEUA at 120 minutes and 180 minutes in the low BMI group and reduced FEUA at 60 minutes in the high BMI group.

**Conclusions:**

These data suggest that BMI influences serum urate and gout risk in response to chronic SSB intake, and renal tubular uric acid handling in response to an acute fructose load. In addition to many other health benefits, avoidance of SSBs may be particularly important in those with overweight/obesity to prevent hyperuricaemia and reduce gout risk.

**Trials registration:**

Australian Clinical Trials Registry ACTRN12610001036000. Registered 24 November 2010.

## Introduction

Serum urate concentrations are influenced by a number of modifiable and non-modifiable factors. Intake of sugar-sweetened beverages is a strong modifiable risk factor for hyperuricaemia and gout [1–4]. The effect of sugar-sweetened beverage intake on serum urate has been attributed to the hepatic effects of fructose on adenosine triphosphate degradation, which in turn, induces urate production [5]. Sugar may also directly interfere with urate excretion via the hexose-uric acid transporter solute carrier family 2, facilitated glucose transporter member 9 (SLC2A9) [4].

Elevated body mass index is another modifiable risk factor for hyperuricaemia and development of gout [6], with a consistent strong relationship between central adiposity and serum urate [7], which may be mediated by the effects of insulin on renal tubular uric acid reabsorption [8]. There are accumulating data that body mass index can modulate the influence of non-modifiable genetic variants on serum urate [9, 10]. The influence of body mass index may also modulate associations of modifiable factors, such as sugar-sweetened beverage intake, with serum urate and gout. The aim of this study was to determine whether the associations between sugar-sweetened beverage intake with serum urate and gout are moderated by body mass index.

## Methods

We addressed the study aim in two separate analyses: first, in a large cross-sectional population study examining the effects of chronic sugar-sweetened beverage intake on serum urate and gout, and second, in a short-term intervention study examining the effects of an acute fructose load on serum urate and fractional excretion of uric acid. In all analyses, the responses were analysed in those with body mass index <25 mg/kg^2^ (low body mass index group) and those with body mass index ≥ 25 mg/kg^2^ (high body mass index group).

### Chronic sugar-sweetened beverage intake serum urate analysis

The effect of chronic sugar-sweetened beverage intake on serum urate in separate body mass index strata was assessed by analysing 12,870 people without gout from the Atherosclerosis Risk in Communities (ARIC), Framingham Heart Study (FHS) and New Zealand (NZ) datasets. The ARIC (n = 8,436) and FHS (n = 3,066) cohorts are US longitudinal studies described by us elsewhere [11] whereas the NZ dataset is a cross-sectional sample set recruited from 2007 to 2014 [4]. The FHS Generation 3 cohort was analysed with all measures taken at examination 1 (2002–2005) and all ARIC measures were taken at examination 1 (1987–1989). Sugar-sweetened beverage consumption was assigned to one of three categories – those drinking no sugar-sweetened beverages (no sugar-sweetened beverage intake group), >0 to <2 sugar-sweetened beverages per day (low sugar-sweetened beverage intake group) and ≥2 sugar-sweetened beverages per day (high sugar-sweetened beverage intake group). Serum urate was regressed against the three categories in body mass index <25 mg/kg^2^, body mass index ≥25 mg/kg^2^ and unstratified. All analyses were adjusted by ethnicity, age, sex, fruit intake, triglycerides, kidney disease (self-reported) and hypertension, and relatedness in FHS. Significant difference in the change in serum urate per sugar-sweetened beverage category between body mass index groups was assessed by calculating a Cochran’s Q test statistic and the corresponding *P* value (*P*_Q_). Data were analysed using STATA version 13.1 (StataCorp, College Station, TX, USA) and R 3.0.2 in the RStudio GUI version 0.98.1087 (R Foundation for Statistical Computing, Vienna, Austria).

### Chronic sugar-sweetened beverage intake gout analysis

The effect of chronic sugar-sweetened beverage intake on gout status in separate body mass index strata was assessed by analysing 2,578 people (n = 1,368 without gout and 1,210 with gout) from the NZ dataset [4]. Gout was clinically ascertained using the 1977 American Rheumatism Association preliminary gout classification criteria [12]. A logistic regression of sugar-sweetened beverage intake (based on the three categories: 0, >0 to <2, >2) with gout status was performed to determine the odds ratio for gout. All analyses were adjusted by ethnicity, age, sex, fruit intake, triglycerides, kidney disease and hypertension. Significant difference in the odds ratios for gout per sugar-sweetened beverage category between body mass index groups was assessed by calculating a Cochran’s Q test statistic and the corresponding *P* value (*P*_Q_). Data were analysed using STATA version 13.1 and R 3.0.2 in the RStudio GUI version 0.98.1087.

### Acute fructose intake analysis

The methods of the acute fructose intake study have been previously reported [13]. Exclusion criteria were: gout, diabetes mellitus or fructose intolerance, diuretic use, fasting glucose >6 mmol/L. Following an overnight fast, 76 healthy volunteers consumed a sugar solution between 8 a.m. and 9 a.m., and blood was obtained prior to ingestion and then 30 minutes, 60 minutes, 120 minutes, and 180 minutes after ingestion. Urine was obtained at each time point for testing of urate and creatinine to allow calculation of fractional excretion of uric acid (uric acid clearance/creatinine clearance expressed as a percentage). Weight and height were measured at the start of the study visit, and body mass index was calculated. The sugar solution of 300 kcal/300 ml was consumed within 10 minutes, according to the protocol of Akhavan and Anderson for fructose-induced hyperuricaemia [14]. This solution contained 80 % fructose and 20 % glucose (64 g fructose and 16 g glucose). The study was approved by the New Zealand Multiregional Ethics Committee, and each participant gave written informed consent.

Data were analysed using a mixed models approach to repeated measures. Significant main and interaction effects were explored using the method of Tukey. Sex, ethnicity and triglycerides were adjusted for within the models. For change in serum urate and other biochemical variables, a mixed models analysis of covariance (ANCOVA) approach to repeated measures was used. For ANCOVA, the dependent variable was change from baseline, and baseline level was included as a covariate. Analyses were performed using SAS v 9.2 (SAS Institute Inc., Cary, NC, USA). *P* <0.05 was considered significant and all tests were two-tailed.

## Results

### Chronic sugar-sweetened beverage intake and serum urate

The characteristics of participants in the chronic sugar-sweetened beverage intake and serum urate analysis are shown in Table 1. The majority of participants were of Caucasian ethnicity (n = 10,443), with smaller numbers of participants of New Zealand Polynesian (n = 918) and African American (n = 1,509) ethnicity. More than half (61.1 %) of participants had a body mass index ≥25 kg/m^2^. The mean sugar-sweetened beverage intake in the low body mass index group was 1.1 drinks/day and in the high body mass index group was 1.2 drinks/day. Serum urate was higher in the high body mass index group compared to the low body mass index group.

**Table 1:** Clinical features of all participants in the chronic sugar-sweetened beverage intake urate analysis

	Percentage with data	All (n = 12,870)	BMI <25 (n = 5,002)	BMI ≥25 (n = 7,862)	*P*
Age, years	100	49.5 (10.1)	48.9 (10.4)	49.9 (9.9)	9 × 10^−9*^
Male sex, n (%)	100	6030 (46.9)	1740 (34.8)	4288 (54.5)	<1.0 × 10^−5**^
NZ East Polynesian dataset, n (%)	100	583 (4.5)	96 (1.9)	487 (6.2)	<1.0 × 10^−5**^
NZ West Polynesian dataset, n (%)	100	271 (2.1)	16 (0.3)	255 (3.2)	
NZ Mixed East/West Polynesian dataset, n (%)	100	64 (0.5)	4 (0.1)	60 (0.8)	
NZ Caucasian dataset, n (%)	100	450 (3.5)	152 (3.0)	298 (3.8)	
ARIC Caucasian dataset, n (%)	100	6927 (53.8)	2930 (58.6)	3996 (50.8)	
FHS Caucasian dataset, n (%)	100	3066 (23.8)	1380 (27.6)	1686 (21.4)	
ARIC African American dataset, n (%)	100	1509 (11.7)	424 (8.5)	1080 (13.7)	
Serum urate, mmol/L	98.4	0.33 (0.09)	0.30 (0.07)	0.36 (0.08)	<1.0 × 10^−300*^
Triglycerides, mmol/L	98.0	1.79 (1.52)	1.45 (1.04)	2.01 (1.72)	<1.0 × 10^−300*^
Body mass index, kg/m^2^	99.9	27.1 (5.4)	22.4 (1.8)	30.1 (4.8)	<1.0 × 10^−300*^
Fruit intake, pieces/day	99.8	1.47 (1.31)	1.45 (1.32)	1.48 (1.31)	0.32^*^
Kidney disease, n (%)	96.3	134 (1.1)	34 (0.7)	100 (1.3)	1.4 × 10^−3**^
Hypertension, n (%)	99.3	1541 (12.1)	395 (7.9)	1143 (14.7)	<1.0 × 10^−5**^

Unless specified, data are presented as mean (SD)

*BMI* body mass index, *NZ* New Zealand, *ARIC* Atherosclerosis Risk in Communities, *FHS* Framingham Heart Study

^*^Two-sample *t* test with unequal variances

^**^Chi-square test

In this analysis, high sugar-sweetened beverage intake was associated with higher serum urate in the entire group (compared with no sugar-sweetened beverage intake, body mass index-adjusted *P* = 0.11 for low sugar-sweetened beverage intake and *P* = 1.9 × 10^−3^ for high sugar-sweetened beverage intake, Table 2). There was no association between sugar-sweetened beverage intake and serum urate in the low body mass index group (*P* = 0.20 for low sugar-sweetened beverage intake and *P* = 0.67 for high sugar-sweetened beverage intake). In contrast, increased sugar-sweetened beverage intake was associated with higher serum urate in the high body mass index group (*P* = 9.4 × 10^−4^ for low sugar-sweetened beverage intake and *P* = 7.2 × 10^−6^ for high sugar-sweetened beverage intake). The difference in serum urate according the sugar-sweetened beverage categories was significantly greater in the high body mass index group than in the low body mass index group (*P*_difference_ = 3.6 × 10^−3^). The difference in serum urate was higher in the high body mass index group for both low sugar-sweetened beverage intake (*P*_difference_ = 1.4 × 10^−3^) and high sugar-sweetened beverage intake (*P*_difference_ = 9.3 × 10^−4^).

**Table 2:** Difference in serum urate concentration for chronic sugar-sweetened beverage intake stratified by body mass index (BMI) group

		Per SSB category		Overall SSB category		
	SSB drinks/day	∆ in serum urate, 95 % CI (mmol/L)	*P* ^**^	∆ in serum urate, 95 % CI (mmol/L)	*P*	*P*_difference_ between BMI <25 and ≥25
All participants (n = 12,081)^*^	0	-	-	0.004 (0.002–0.006)	9.7 × 10^−4^	-
	>0 to <2	0.004 (0.000–0.008)	0.11			
	≥2	0.009 (0.005–0.014)	1.9 × 10^−3^			
BMI <25 (n = 4,731)	0	-	-	−0.000 (−0.004– 0.003)	0.97	3.6 × 10^−3^
	>0 to <2	−0.004 (−0.010–0.002)^†^	0.20			
	≥2	−0.002 (−0.009–0.006)^††^	0.67			
BMI ≥25 (n = 7, 350)	0	-	-	0.007 (0.004–0.010)	1.4 × 10^−5^	
	>0 to <2	0.010 (0.004–0.015)^†^	9.4 × 10^−4^			
	≥2	0.015 (0.008–0.021)^††^	7.2 × 10^−6^			

The difference in serum urate in the overall sugar-sweetened beverage (SSB) category is the average difference from sugar-sweetened beverage category 1 to category 2 to category 3 (i.e. 0, to >0 to <2, to ≥2). All analysis adjusted by sample set (Atherosclerosis Risk in Communities (ARIC) Caucasian, Framingham Heart Study (FHS) Caucasian, New Zealand (NZ) Caucasian, East Polynesian, West Polynesian, Mixed East Polynesian/West Polynesian, ARIC African American), age, sex, fruit intake (continuous variable), kidney disease, hypertension, triglycerides, and relatedness

^*^Analysis adjusted by BMI

^**^Compared with referent group (0 SSB/day)

^†^ *P*_difference_ between BMI groups =1.4 × 10^−3^

^††^ *P*_difference_ between BMI groups = 9.3 × 10^−4^

### Chronic sugar-sweetened beverage intake and gout status

The characteristics of participants in the chronic sugar-sweetened beverage intake and gout analysis are shown in Table 3. The majority of participants with gout (76.8 %) were on urate-lowering therapy. There were differences between cases and controls with respect to sex, age, ethnicity, serum urate, triglycerides, body mass index, and history of hypertension and kidney disease (Table 3).

**Table 3:** Clinical features of participants in the gout analysis

	Percentage with data	All (n = 2,578)	Controls (1,368)	Cases (1,210)	*P*
Age, years	100	52.1 (16.4)	47.0 (16.5)	57.8 (14.4)	4.9 × 10^−66*^
Male sex, n (%)	100	1739 (67.5)	740 (54.1)	999 (82.6)	<1.0 × 10^−5**^
NZ East Polynesian dataset, n (%)	100	901 (35.0)	583 (42.6)	319 (26.4)	<1.0 × 10^−5**^
NZ West Polynesian dataset, n (%)	100	597 (23.2)	271 (19.8)	326 (26.9)	
NZ Mixed East/West Polynesian dataset, n (%)	100	92 (3.6)	64 (4.7)	28 (2.3)	
NZ Caucasian dataset, n (%)	100	987 (38.3)	450 (32.9)	537 (44.4)	
Serum urate at the time of recruitment, mmol/L	86.5	0.39 (0.11)	0.37 (0.10)	0.43 (0.11)	5.9 × 10^−37*^
Triglycerides, mmol/L	86.9	2.14 (1.4)	1.93 (1.18)	2.39 (1.60)	2.3 × 10^−14*^
Body mass index, kg/m^2^	100	32.4 (7.6)	31.3 (7.5)	33.6 (7.6)	2.6 × 10^−15*^
Fruit intake, pieces/day	98.9	2.9 (4.6)	2.8 (4.2)	3.0 (5.0)	0.27^*^
Kidney disease, n (%)	98.0	295 (11.7)	57 (4.3)	238 (20.1)	<1.0 × 10^−5**^
Hypertension, n (%)	98.3	975 (38.5)	309 (23.0)	666 (55.9)	<1.0 × 10^−5**^

Unless specified, data are presented as mean (SD)

*NZ* New Zealand

^*^Two-sample *t* test with unequal variances

^**^Chi-square test

In the low body mass index group, after adjustment for all these variables, low sugar-sweetened beverage intake was associated with a reduced odds ratio (OR) for gout (OR 0.43, *P* = 0.043) compared to no intake, and there was no increase in the odds ratio for high sugar-sweetened beverage intake (OR 0.64, *P* = 0.31) (Table 4). In contrast, high sugar-sweetened beverage intake was associated with a higher odds ratio for gout (OR 1.33, *P* = 0.033) in the high body mass index group. The odds ratios according to the sugar-sweetened beverage categories were significantly greater in the high body mass index group than in the low body mass index group (*P*_difference_ = 0.012). The difference in the odds ratios for gout were significantly different between the body mass index groups for both low sugar-sweetened beverage intake (*P*_difference_ = 5.9 × 10^−4^) and high sugar-sweetened beverage intake (*P*_difference_ = 0.028).

**Table 4:** Difference in risk of gout for chronic sugar-sweetened beverage intake stratified by body mass index (BMI) group

		Per SSB category		Overall SSB category		
	SSB drinks/day	Odds ratio (95 % CI)	*P* ^**^	Odds ratio (95 % CI)	*P*	*P*_difference_ between BMI <25 and ≥25
All participants (n = 2,144)^*^	0	-	-	1.09 (0.96–1.23)	0.19	-
	>0 to <2	0.83 (0.64–1.07)	0.15			
	≥2	1.20 (0.93–1.54)	0.16			
BMI <25 (n = 304)	0	-	-	0.74 (0.49–1.13)	0.16	0.012
	>0 to <2	0.43 (0.19–0.97)^†^	0.043			
	≥2	0.64 (0.28–1.50)^††^	0.31			
BMI ≥25 (n = 1,840)	0	-	-	1.15 (1.01–1.32)	0.035	
	>0 to <2	0.92 (0.70–1.21)^†^	0.55			
	≥2	1.33 (1.02–1.74)^††^	0.033			

The difference in gout risk in the overall sugar-sweetened beverage (SSB) category is the average difference from sugar-sweetened beverage category 1 to category 2 to category 3 (i.e. 0, to >0 to <2, to ≥2). All analysis adjusted by age, sex, fruit intake (continuous variable), kidney disease, hypertension, triglycerides, and ethnicity (New Zealand (NZ) Caucasian, East Polynesian, West Polynesian and Mixed East/West Polynesian)

^*^Analysis adjusted by BMI

^**^Compared with referent group (0 SSB/day)

^†^ *P*_difference_ between BMI groups =5.9 × 10^−4^

^††^ *P*_difference_ between BMI groups = 0.028

### Acute fructose intake

The characteristics of participants in the acute fructose intake analysis are shown in Table 5. As previously reported, the study participants were of European (n = 25), Māori (n = 25) and Pacific (n = 26) ethnicity. More than half (59 %) of participants had a body mass index ≥25 kg/m^2^. There were fewer European and more Pacific participants in the high body mass index group. All analyses were adjusted for sex and ethnicity.

**Table 5:** Clinical features of participants in the acute fructose loading study

	All (n = 76)	BMI <25 (n = 31)	BMI ≥25 (n = 45)	*P*
Age, years	30 (17)	28 (14)	33 (19)	0.22
Male sex, n (%)	45 (59 %)	15 (48 %)	30 (67 %)	0.18
European ethnicity, n (%)	25 (33 %)	16 (52 %)	9 (20 %)	0.009
Māori ethnicity, n (%)	25 (33 %)	11 (35 %)	14 (31 %)	0.88
Pacific ethnicity, n (%)	26 (34 %)	4 (13 %)	22 (49 %)	0.002
Serum urate, mmol/l	0.36 (0.11)	0.31 (0.09)	0.40 (0.11)	0.0003
Triglycerides, mmol/L	1.24 (0.78)	0.93 (0.34)	1.44 (0.92)	0.0013
Fractional excretion of uric acid, %	5.82 (2.15)	5.97 (2.46)	5.70 (1.97)	0.60
Body mass index, kg/m^2^	27.4 (5.6)	22.6 (1.66)	30.8 (4.9)	<0.0001
Kidney disease, n (%)	1 (1.3 %)	1 (3.2 %)	0 (0 %)	0.82
Hypertension, n (%)	6 (7.9 %)	2 (6.5 %)	4 (8.9 %)	0.99

Unless specified, data are presented as mean (SD)

*BMI* body mass index

**Fig. 1:**
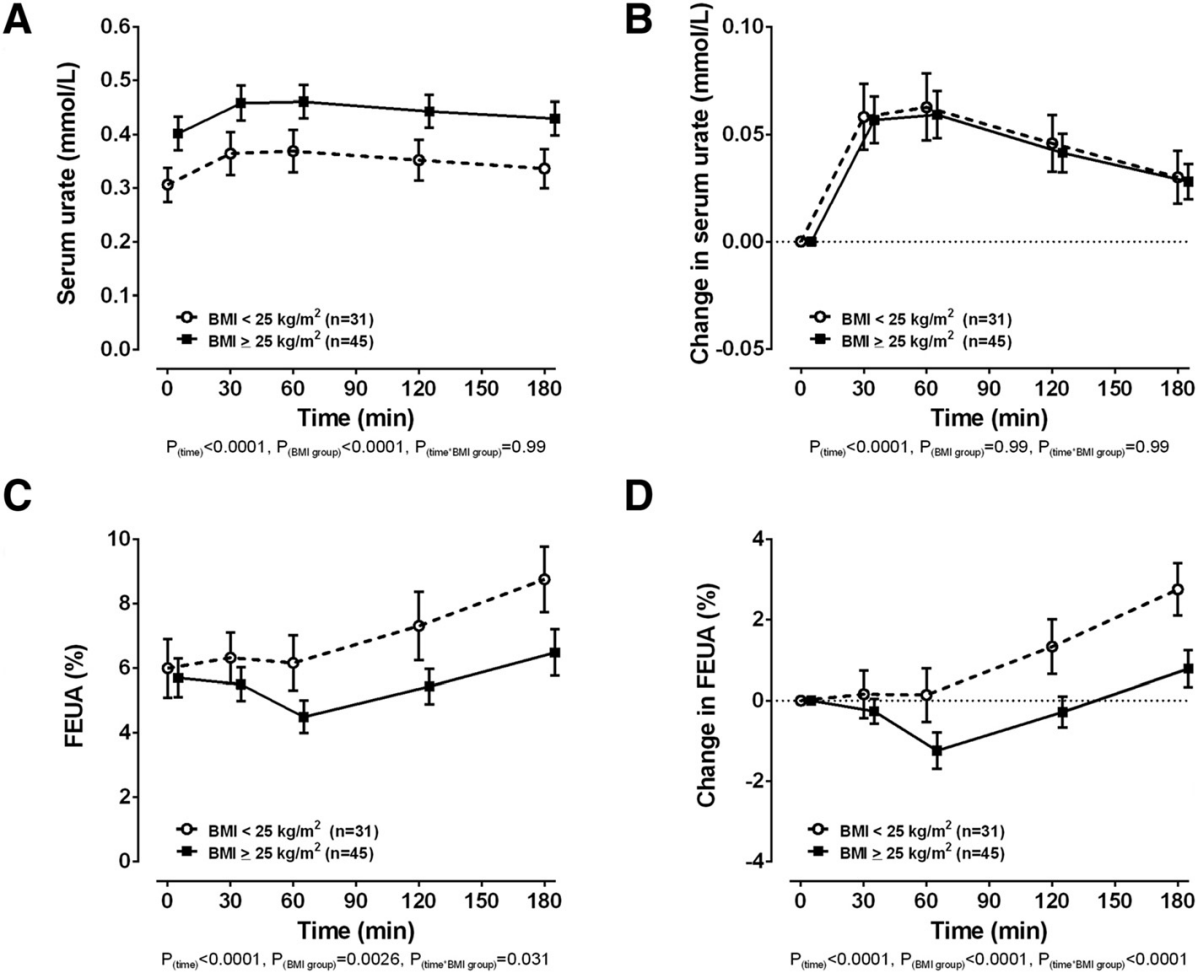
The effect of body mass index (BMI) group on serum urate and fractional excretion of uric acid (FEUA) during an acute fructose load. **a** Serum urate concentration. **b** Change in serum urate concentration. **c** FEUA. **d** Change in FEUA. Data are presented as mean (95 % CI). Sex, ethnicity and triglyceride-adjusted analysis of covariance (ANCOVA) *P* values are shown throughout

In the acute fructose feeding study, serum urate was higher in the high body mass index group at baseline and throughout the observation period (Fig. 1a, *P*_body mass index group_ <0.0001). There were similar acute serum urate increases in both body mass index groups in response to the fructose load (Fig. 1b, *P*_interaction_ = 0.99). The fractional excretion of uric acid was similar between the two body mass index groups at baseline (Fig. 1c, Tukey-Kramer adjusted *P* = 1.0). The fractional excretion of uric acid in the low body mass index group increased significantly from baseline at 120 minutes and 180 minutes after the fructose load (Fig. 1c and d, Tukey-Kramer adjusted *P* = 0.003 and <0.0001 respectively). In contrast, the fractional excretion of uric acid in the high body mass index group decreased significantly from baseline at 60 minutes after the fructose load (Fig. 1c and d, Tukey-Kramer adjusted *P* = 0.0002), and no significant increase in fractional excretion of uric acid from baseline was observed even after 180 minutes of fructose intake. The fractional excretion of uric acid responses in the body mass index groups differed significantly following the fructose load (Fig. 1c and d, *P*_interaction_ <0.0001).

## Discussion

This study has shown that body mass index influences urate homeostasis associated with sugar-sweetened beverage intake. With low body mass index, chronic sugar-sweetened beverage intake is associated with negligible difference in serum urate. This may be due to the ability of the renal tubule to rapidly increase uric acid excretion following an event that increases serum urate, thus normalising serum concentrations. In contrast, for those with high body mass index, chronic sugar-sweetened beverage intake is associated with large increases in serum urate. With high body mass index, the renal tubular urate handling response to the acute fructose is altered, with reduction in fractional excretion of uric acid and slow renal clearance of uric acid. In the context of chronic intake of dietary sugar, these renal responses may lead to prolonged serum urate elevations.

Consistent with the serum urate results, we observed that high sugar-sweetened beverage intake was associated with a higher odds ratio for gout in those with high body mass index, but not in normal weight individuals. Due to the high use of urate-lowering therapy in participants with gout, we were unable to determine whether people with gout and high body mass index have an amplified serum urate response to sugar-sweetened beverage intake, compared to normal weight people with gout. However, our gout analysis does suggest that the observed serum urate interaction between chronic sugar-sweetened beverage intake and BMI observed in the general population is clinically relevant.

In contrast to the in-hospital study by Yamashita et al. reporting lower fractional excretion of uric acid (FEUA) in people with obesity compared to normal weight control participants [15], baseline FEUA were similar between BMI groups in our acute feeding study, which studied community-dwelling healthy volunteers. However, acute FEUA responses were substantially different following the fructose load. Although higher serum urate was observed in the high body mass index group throughout the acute feeding study, we did not observe differences between body mass index groups in change in serum urate following a fructose load. This is perhaps surprising given the chronic sugar-sweetened beverage intake study and the acute fructose study fractional excretion of uric acid results. These data suggest that the hepatic urate production response to an acute fructose load is not influenced by body mass index. In the context of chronic sugar-sweetened beverage intake, the ability of the renal tubule to clear urate following hepatic synthesis may account for the differential serum urate responses observed between the chronic and acute study. Unfortunately, there are currently no large publicly available databases with fractional excretion of uric acid data to further clarify whether the renal responses to chronic fructose intake differ between different body mass index groups. Differential serum urate responses to acute and chronic sugar-sweetened beverage intake have been reported previously in relation to genetic risk factors such as *SLC2A9* [4, 13], and it is possible that chronic sugar-sweetened beverage exposure influences other pathways that regulate serum urate.

## Conclusions

Consistent with recent reports showing that body mass index influences non-modifiable genetic associations with serum urate [9, 10], this study shows that chronic sugar-sweetened beverage intake is associated with elevated serum urate and gout status in those with high body mass index, with minimal effect observed in lean individuals. In addition to many other health benefits, avoidance of sugar-sweetened beverages may be particularly important in those with high body mass index to prevent hyperuricaemia and reduce gout risk.

## Abbreviations

ANCOVA: analysis of covariance
ARIC: Atherosclerosis Risk in Communities
BMI: body mass index
FEUA: fractional excretion of uric acid
FHS: Framingham Heart Study
NZ: New Zealand
OR: odds ratio
SLC2A9: transporter solute carrier family 2, facilitated glucose transporter member 9
SSB: sugar-sweetened beverage
US: United States
